# No Myocardial Vulnerability to Mental Stress in Takotsubo Stress Cardiomyopathy

**DOI:** 10.1371/journal.pone.0093697

**Published:** 2014-04-02

**Authors:** Olov Collste, Per Tornvall, Örjan Sundin, Mahbubul Alam, Mats Frick

**Affiliations:** 1 Departments of Clinical Sciences and Education, Södersjukhuset, Karolinska Institutet, Stockholm, Sweden; 2 Departments of Clinical Sciences, Danderyd Hospital, Karolinska Institutet, Stockholm, Sweden; 3 Department of Psychology, Mittuniversitetet, Östersund, Sweden; Temple University, United States of America

## Abstract

**Objectives:**

Due to the frequent use of coronary angiography the awareness of Takotsubo stress cardiomyopathy (TSC) has increased although the exact pathophysiology of TSC is still largely unknown. Our objective was to investigate the effects of mental stress on myocardial function, heart rate variability (HRV) and salivary cortisol (SC) in TSC patients.

**Design:**

This study is a case-control study and a sub-study of the Stockholm Myocardial Infarction with Normal Coronaries (SMINC) study.

**Setting:**

Mental stress test was performed more than 6 months after the acute event in TSC patients and age- and sex-matched controls. Standard echocardiography and tissue Doppler imaging (TDI) - derived time-phases of cardiac cycle were recorded to calculate myocardial performance index (MPI) to assess ventricular function before and during mental stress. Holter-ECG recording was made to estimate HRV before, during and after mental stress. SC was measured at baseline, before and 20 minutes after mental stress.

**Subjects:**

Twenty-two TSC patients and 22 sex-and age-matched controls were recruited from the SMINC-study and investigated with a mental stress test. All TSC patients had a previous normal cardiovascular magnetic resonance investigation.

**Results:**

There were no significant differences at rest or during mental stress for left and right ventricular MPI or other standard diastolic variables between TSC patients and controls. HRV did not differ between TSC patients and controls. There was a trend towards less increase in SC after mental stress in TSC patients compared to controls.

**Conclusion:**

Mental stress did not induce a significant difference in myocardial function or HRV response between TSC and controls. Moreover, no significant difference could be seen in SC response at baseline, during or after mental stress. This study indicates that myocardial vulnerability to mental stress does not persist in TSC patients.

## Introduction

Takotsubo stress cardiomyopathy (TSC) is an important subgroup of myocardial infarction with normal coronary arteries (MINCA). In a recent study, it was shown that MINCA occured in as many as 7–8% of all patients with acute coronary syndrome [1]. There is evidence of a strong relationship between TSC and acute mental or physiological stress [2]. Case studies have reported TSC occurring during dobutamine stress echocardiography (DSE) [3], [4]. However, a recent study on the effect of DSE in patients with a previous episode of TSC could not show a difference in tissue doppler imaging (TDI) variables during stress [5]. Although most episodes of TSC are precipitated by mental stress [6] the effect of mental stress on left and right ventricular function in TSC patients after the acute event is unknown. Using different time phases of cardiac cycle as a means of myocardial function, myocardial performance index (MPI) gives an estimate of the overall systolic and diastolic ventricular function. Since MPI is a sensitive marker of ventricular function it is valuable in evaluating effects of mental stress [7].

A recent study of heart rate variability (HRV) in TSC showed that standard deviation of all normal intervals (SDNN) was significantly lower in the TSC group compared with controls in the sub-acute phase [8]. However, the effect of mental stress on HRV in TSC patients after the acute event has not been studied previously.

Since stress is involved in TSC triggering it is reasonable to investigate whether cortisol levels during stress are different in TSC in comparison to a control group. Salivary cortisol (SC) reflects levels of free cortisol in blood. Given the ease of collecting saliva, it is an established method in stress research [9].

In order to examine the pathophysiology of TSC this study aimed to study ventricular function, HRV and SC in TSC patients and their response to mental stress in comparison with a sex- and age-matched control group. Our hypothesis was that TSC patients have an increased vulnerability to mental stress after discharge from their index admission for TSC.

## Methods

### Ethical Considerations

This study was performed in accordance with the Declaration of Helsinki and good clinical practice. The study was approved by the Regional Ethical Review Board in Stockholm. A written consent was acquired from the study participants in the main study but oral consent was acquired for this sub-study [1]. This consent procedure was approved by the Regional Ethical Review Board in Stockholm. Since a written consent was not mandatory, oral consent was achieved and documented in the electronical medical case notes.

### Study Group

Twenty-two patients with a previous episode of TSC and 22 sex- and age-matched controls were recruited from the Stockholm Myocardial Infarction with Normal Coronaries (SMINC) study. The controls were not investigated with coronary angiography but had no signs or symptoms of coronary artery disease and a normal exercise stress test prior to inclusion in the study. For the HRV data 20 TSC patients and 18 controls were studied. The HRV part of the study started after the TDI and SC parts. Hence, the lower number of TSC patients and controls examined with HRV.

All patients and controls were investigated with a mental stress test. For TSC patients the investigation was performed more than 6 months after the acute event. All TSC patients were previously investigated with cardiovascular magnetic resonance (CMR) imaging, during the sub-acute phase, and found to have no signs of myocardial infarction (myocardial necrosis) or myocarditis. If the TSC patient or control were on beta-blockers the treatment was withheld on the day of examination with mental stress.

### Mental Stress Test

The mental stress test consisted of two parts. The first part was an anger recall interview [10], [11] where the patient or control was asked to recall an upsetting situation during the last few months prior to the investigation and then speak about that situation for a few minutes. A few follow-up questions were asked by the interviewer about this upsetting situation. The second part of the mental stress was a mental arithmetics task [10], [12] that was performed directly after the anger recall interview. The patient or control was asked to subtract 7 from 200 in continuing steps as fast as possible. This test was done with increased stress by the interviewer.

### Echocardiography

For the echocardiograms a Philips (Amsterdam, Netherlands) iE33 was used. Echocardiography was performed twice, at baseline and during the psychological stress. Recording and calculations of different cardiac dimensions and standard functions were done according to the recommendations of the European Society of Echocardiography [13]. Recording of myocardial velocities were performed using TDI as described before [5]. From apical views, myocardial veclocities were recorded at four LV sites near the mitral annulus representing septal, lateral, anterior and inferior sites of the LV and at one site at the RV near the tricuspid annulus. From these velocity recordings different time intervals of the cardiac cycle was calculated. MPI was calculated by measuring the isovolumetric contraction time (ICT), isovolumetric relaxation time (IRT) and ejection time (ET). MPI was calculated from (ICT+IRT)/ET. A mean value from four LV sites was used to assess global LV-MPI. For the RV, only the lateral site was used to calculate RV-MPI. In addition, E/E′ was also calculated (the ration between early transmitral Doppler velocity and early diastolic myocardial velocity - an expression of LV filling pressure). Three consecutive cycles were used to calculate all echo-Doppler parameters. During mental stress LV-MPI, RV-MPI, E/E′ and E/A were once again assessed. The images obtained with echocardiography were analyzed in Syngo Dynamics software (Siemens Healthcare, Erlangen, Germany).

### Intra-observer Variability

All echocardiographic data were analyzed by an experienced echocardiographer. Data were then analyzed a second time blindly by the same echocardiographer. Data sets acquired were used for calculation of intra-class correlation coefficient (ICC) to estimate intra-observer variability. Intra-observer variability was 0.922 and 0.874 for LV-MPI and RV-MPI, respectively, in baseline echocardiographic data. During mental stress ICC decreased to 0.898 and 0.737, respectively. Complete intra-observer variability data are shown in Table 1.

**Table 1:** Intra-observer variability for tissue Doppler data.

	Intra-observer variability at rest	Intra-observer variability during stress
E/A	0.949	0.889
DT	0.451	n.a.
E/E′	0.745	0.788
LV-MPI	0.922	0.898
RV-MPI	0.874	0.737

Intra-observer variability for different echocardiographic variables expressed as intraclass correlation coefficient for single measures. Median values. E/A; early and atrial transmitral flow fraction, E/E′; early transmitral flow and early tissue Doppler velocity flow fraction, MPI; myocardial performance index, RV-MPI; right ventricular MPI. N.a.; not applicable.

### Holter-ECG

The electrocardiographic (ECG) data were collected with Novacor Vista Plus (Ruell, Malmaison, France) Holter-ECG system. Collecting ECG data began at rest 10 minutes before psychological stress and continued during psychological stress and until at least 10 minutes after the mental stress test was finished. HolterSoft (Novacor, Ruell, Malmaison, France) was used to automatically analyze the HRV of the collected ECG data. The time domain variables SDNN and standard deviation of the average of all normal intervals (SDANN) were analyzed in addition to analysis of mean-, minimum- and maximum heart rate. ECG-recordings were also analyzed pre-stress, during stress and post-stress separately. The mean-, minimum- and maximum heart rate were apart from the automatic analysis also visually interpreted to ensure an accurate estimate of the variables.

### Salivary Cortisol

Salivary cortisol (SC) was collected at five separate occasions using an absorbent pad. Basal SC was collected by the patient or control after waking-up (around 08.00 a.m.), 30 minutes after waking-up and before going to bed. Additionally SC was collected before and 20 minutes after the mental stress test. The mental stress test was performed around five o′clock in the afternoon in all cases. SC test tubes were kept in a refrigerator before being sent to laboratory for analysis. The laboratory analysis used was a competitive radio-immuno assay, including a polyclonal antibody coated tube (Spectria Cortisol RIA, Orion Diagnostica, Espoo, Finland).

### Statistics

The primary end-point of the study was MPI at stress. By including 22 patients and 22 controls in the respective groups the study had a power of 80% to detect a difference in MPI of 20% (p<0.05). Non-parametric Mann-Whitney U-test was used to compare median values for the two independent groups. Interquartile range (IQR) was calculated for the main outcome variables. Wilcoxon Signed Rank test was used to compare the median value within each group. Intra-class correlation coefficient was used to calculate intra-observer variability. Statistical analysis was made with SPSS version 22 (IBM, Armonk, New York, NY, USA). A p-value of <0.05 was considered significant.

## Results

### Baseline Characteristics

The mean age of the TSC group was 63.2 years and for the control group 63.6 years. In the TSC group the prevalence of hypertension was 55% while it was 27% in the control group. Two patients in the TSC group, but none in the control group, had diabetes mellitus. Treatment with beta-blockers and calcium channel blockers were more frequent in TSC patients. Baseline characteristics for both groups are shown in Table 2.

**Table 2:** Baseline characteristics.

	Takotsubo	Control
Number	22	22
Age (mean)	63.2	63.6
Gender (male/female)	21-Jan	21-Jan
Present smoker	27%	0%
Diabetes	9%	5%
Hypertension	55%	27%
Normal ECG	86%	96%
Beta-blocker	64%	9%
Calcium blocker	18%	0%
ACE-inhibitor	18%	18%

ECG; Electrocardiography, ACE; Angiotensin converting enzyme.

### Mental Stress Test

All TSC patients and controls completed the mental stress test. At rest the heart rate was 58 beats per minute (bpm) and 60 bpm for the TSC patients and controls, respectively. During peak stress they reached 89 and 94 bpm, respectively (p = 0.28). Self-estimated acute stress level (Likert-type scale 0–6) during mental stress was 2.7 and 2.6 for TSC patients and controls, respectively (p = 0.67). TSC patients estimated that their stress level was 4.4 on the same Likert-type scale during the acute event.

### Echocardiographic Results

All TSC patients and controls had normal left- and right ventricular function. LV-MPI and RV-MPI did not differ significantly between TSC patients and controls at rest or during mental stress, Tables 3 and 4. During peak stress LV-MPI was 0.59 and 0.53, respectively (p = 0.53). There were no significant differences between TSC patients and controls in E/A, DT or E/E′ at rest or during stress.

**Table 3:** Echocardiographic data at rest.

	Takotsubo	Control	p-value
E/A	0.97 [0.49–1.45]	1.11 [0.69–1.53]	0.57
DT	201 [167–235]	183 [139–227]	0.43
E/E′	8.2 [5.5–10.9]	8.0 [5.4–10.5]	0.34
LV-MPI	0.54 [0.40–0.68]	0.54 [0.45–0.63]	0.98
RV-MPI	0.36 [0.21–0.51]	0.37 [0.31–0.43]	0.98

Median values [interquartile range]. E/A; early and atrial transmitral flow fraction, DT; deceleration time, E/E′; early transmitral flow and early tissue Doppler velocity fraction, LV-MPI; left ventricular myocardial performance index, RV-MPI; right ventricular MPI. The non-parametric Mann-Whitney U-test was used to compare the median values for the two independent groups.

**Table 4:** Echocardiographic data during psychological stress.

	Takotsubo	Control	p-value
E/A	0.90 [0.56–1.24]	1.01 [0.82–1.20]	0.21
E/E′	8.9 [6.9–10.9]	8.6 [6.6–10.6]	0.21
LV-MPI	0.59 [0.46–0.72]	0.53 [0.48–0.58]	0.53
RV-MPI	0.42 [0.27–0.57]	0.40 [0.27–0.53]	0.24

Median values [interquartile range]. E/A; early and atrial transmitral flow fraction, E/E′; early transmitral flow and early tissue Doppler velocity flow fraction, MPI; myocardial performance index, RV-MPI; right ventricular MPI.

### Heart Rate Variability

**Figure 1:**
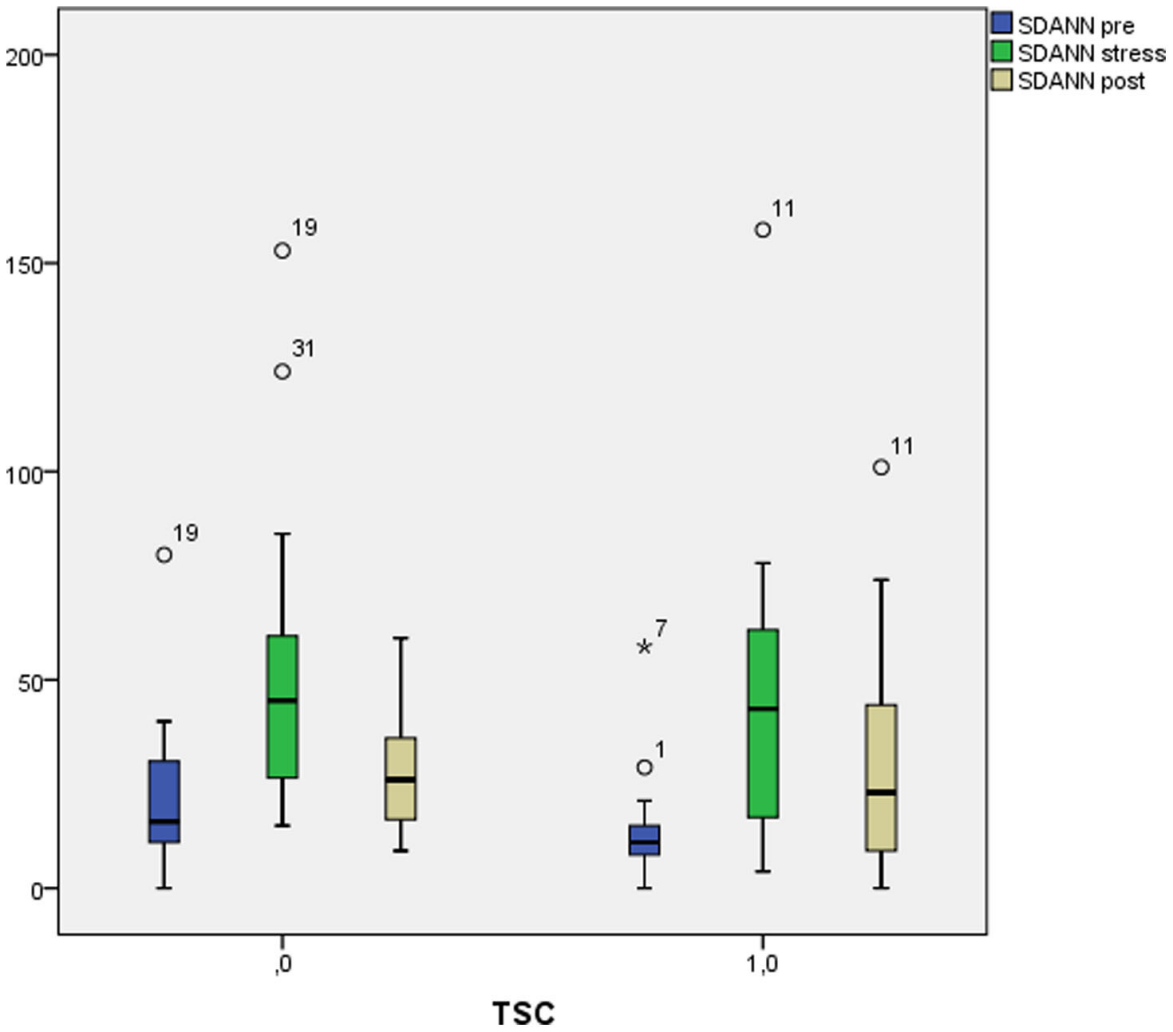
Results of heart rate variability. Horisontal thick line; median value, Boxes; interquartile range, Whiskers; 95% confidence interval. TSC; Takotsubo stress cardiomyopathy, SDANN; standard deviation of the average normal (NN) intervals measured between consecutive sinus beats.

The duration of Holter-ECG was 32 minutes and 30 minutes for the TSC patients and controls, respectively. Overall SDNN was 61 and 76 ms for TSC patients and controls, respectively (p = 0.21) while SDANN was 41 and 47 ms, respectively (p = 0.19). SDANN pre-stress was 11 ms and 16 ms for TSC patients and controls, respectively (p = 0.11). Both groups increased significantly in SDNN from pre-stress to during stress (p = 0.002). Excluding beta-blocker treated TSC patients or controls there was a trend towards greater increase in SDNN from pre-stress to during stress in the TSC group (p = 0.055). There was no significant difference between the groups post-stress. See Table 5 and Figure 1 for complete Holter-ECG results.

**Table 5:** Results of heart rate variability analysis.

	Takotsubo	Controls	p-value
SDNN	61 [30–92]	76 [38–114]	0.21
SDNN pre	45 [17–73]	49 [22–77]	0.38
SDNN stress	65 [30–100]	64 [28–100]	0.5
SDNN pre-stressΔ	22 [−14–53]	20 [−21–64]	0.002*
SDNN post	49 [14–83]	56 [24–87]	0.25
SDANN	41 [11–70]	47 [20–73]	0.19
SDANN pre	11 [3]–[19]	16 [−5–27]	0.11
SDANN stress	43 [−5–90]	45 [10–81]	0.74
SDANN post	23 [−18–64]	26 [5–47]	0.65
SDNN pre-stressΔ no BB	39 [6–72]	18 [−18–54]	0.055

Median values in milliseconds [interquartile range]. SDNN; standard deviation of all normal (NN) intervals measured between consecutive sinus beats, SDANN; standard deviation of the average NN intervals, Pre, stress and post; pre-stress, during stress and post-stress measurements, BB; beta-blocker, *p-value for difference within each group.

The non-parametric Mann-Whitney U-test was used to compare the median values for the two independent groups. Wilcoxon Signed Rank test was used to compare the median value within each group.

### Salivary Cortisol

The second basal SC measurement, taken 30 minutes after waking-up, was 12.4 and 17.3 nmol/L in TSC patients and controls, respectively (p = 0.25). The difference in SC before and after mental stress was −0.6 and 0.6 for TSC patients and controls, respectively (p = 0.099). In all other measurements no significant differences could be seen between the groups (see Table 6).

**Table 6:** Results of salivary cortisol.

	Takotsubo	Control	p-value
BAS1	11.4 [1.4–21.4]	11.3 [0.5–22.1]	0.85
BAS2	12.8 [−1.1–26.7]	17.3 [4.6–30.0]	0.25
BAS3	2.1 [−1.3–5.5]	1.3 [0.2–2.4]	0.31
MST1	4.6 [−0.8–10.0]	4.9 [0.9–8.9]	0.29
MST2	3.9 [−1.5–9.3]	4.4 [0.5–8.3]	0.88
MSTΔ	−0.6 [−3.6–2.35]	0.6 [−2.5–3.6]	0.099

Median values in nmol/L [interquartile range]. BAS1; when waking-up, BAS2; 30 minutes after waking-up, BAS3; before going to bed, MST1; before mental stress test, MST2; 20 minutes after mental stress test, MSTΔ; MST2 minus MST1.

## Discussion

To our knowledge this is the first study of TDI, HRV and SC at mental stress performed after the acute event in TSC patients. Contrary to our hypothesis that TSC patients have an increased vulnerability to mental stress after the acute event, we found no significant differences in left and right ventricular function (e. g. TDI parameters), in HRV or SC levels between TSC patients and controls.

In order to evaluate myocardial performance we used MPI. Previously changes in MPI have been shown to correlate with mortality and an increased incidence of heart failure [14]. MPI can be derived both from Doppler flow and from TDI measurements [15] where the former is the original version used by Tei et al. [16]. Compared to pulsed-wave Doppler used by Tei et al. MPI derived from tissue Doppler has been shown to correlate better with LV ejection fraction and functional capacity [17]. Since MPI is an estimate of systolic as well as diastolic ventricular function it has been shown to be a sensitive marker for ventricular function [7]. Previous case reports of TSC have shown that both the left and right ventricle have been affected, hence, it was of interest to study not only LV-MPI but also RV-MPI [18]–[20].

In this study, which was performed approximately two years after the acute TSC event, we found a normalization of LV-MPI and RV-MPI at rest. This might indicate an expression of a slow recovery and normalization of left- and right ventricular function visible with TDI. Moreover, during peak mental stress there were no differences in LV-MPI and RV-MPI between the groups, which further strengthen the normalization of left- and right ventricular function in TSC patients over time and also support the hypothesis that recurrence of TSC is relatively rare [6].

HRV with low SDNN has previously been linked to increased mortality after acute myocardial infarction [21] and increased mortality in the elderly [22]. Recently HRV in TSC was studied and compared with controls [8]. That study was done after the acute stressful event during admission to hospital by recording of a 24-hour ECG. It was shown that SDNN was significantly lower in the TSC group compared with controls, although normalized after a few weeks (mean 23 days after admission to hospital) on a second 24-hour ECG. However, the effect of a mental stress test on TSC patients has not been studied previously.

In our study we found no significant differences in overall HRV during mental stress between TSC patients and controls. In addition an analysis of HRV pre-stress, during stress and post-stress did not show any significant differences between the groups.

Although no mental stress test study has been performed on TSC patients and controls previously, the effect of mental stress on HRV has been extensively studied. In patients with coronary artery disease (CAD) an increase in HRV was absent during mental stress in comparison to a control group [23]. However, several factors affect HRV; cigarette smoking has been linked to low HRV [24], beta-blockers and ACE-inhibitors increase HRV [25], [26] and, diabetes mellitus and hypertension has been shown to decrease HRV [27], [28]. These studies demonstrate that differences in baseline characteristics are crucial to consider while considering HRV results.

Baseline characteristics in our study might have affected HRV in different ways. The TSC group had a higher percentage of hypertension and present smokers which may decrease HRV. On the other hand, treatment with beta-blockers and ACE inhibitors raises HRV and these treatments were more prevalent in the TSC group than in the control group. In the study by Krstacic et al. HRV was normalized three weeks after the acute event in TSC patients [8]. Despite extensive treatment with beta-blockers among TSC in their study, as well as in ours, we found a larger absolute difference in HRV numbers compared to what they found in the sub-acute phase of TSC.

Previous studies on HRV and acute stress have showed a decrease in SDNN using Stroop word color conflict test and cold pressor test, respectively [29], [30]. In this study we found an increase in HRV during stress for both TSC patients and controls. The reason for this could possibly be found in the amount of stress achieved in this study. We can speculate that when a substantial amount of stress is put on a TSC patient or control the resting mixture of parasympathetic and sympathetic influence is trifling. Instead sympathetic influence is almost completely dominant, hence, a different pattern emerges when measuring HRV.

In order to evaluate the effect of beta-blockers we performed an additional analysis with exclusion of TSC patients and controls who had ongoing treatment with beta-blockers. In this analysis we noted an accentuated increase in SDNN from rest to peak among the TSC patients. Hence, there was a trend towards TSC patients having a lower HRV at rest and a greater HRV during stress. This could indicate an altered sympathetic activity in TSC patients compared to controls. However, the TSC group without beta-blockers was small so this sub-analysis should be treated with caution, and to what extent TSC patients have an altered HRV during mental stress, remains to be settled.

Although the effect of mental stress on cortisol in TSC patients has not been studied previously it has been performed in patients with CAD. Nijm et al. showed that, by using mental arithmetics, there was a significantly blunted SC response in CAD patients compared to controls [31]. This was achieved although the heart rate increased less than 10% during the mental stress test. In our study we achieved a more than 50% increase in heart rate during the mental stress test without a significant effect on SC. The effect on heart rate has to be considered an excellent mental stress test on the basis of what has been reported previously [10], [31]. Compared to the target heart rate of >85% in for example DSE it may still be sub-optimal when detecting differences in TDI, HRV or SC [32]. However, in comparison with earlier studies of mental stress an increase of around 50% in heart rate should be sufficient. Moreover, pharmacological stress is separate from mental stress and may thereby have different effects on TDI, HRV and SC despite a lower heart rate.

There are caveats using SC as a method for measuring the effect of mental stress. One is that around thirty percent of SC is enzymatically converted to cortisone in saliva [9]. However, given the ease of collecting SC and that is has previously been shown to have a flattened diurnal response in patients with known CAD [31], SC is probably still the method of choice for this type of research.

After mental stress we noted a trend towards decreased levels of SC in TSC patients compared to controls. This may reflect a real difference where TSC patients have a decreased SC in response to mental stress. The same difference, albeit weaker, could be seen in absolute numbers in the second basal cortisol level. However, despite a relatively high stress level we could not show any significant differences between TSC patients and controls in SC response.

There are many different mental stress test methods to choose from [10], [33]. All methods have their advantages and disadvantages. It is also crucial how the mental stress test is performed. Our methods of choice were anger recall interview and mental arithmetics [10]. We choose them because they were feasible methods to perform, reasonably validated and because they had been used in studies on patients with CAD [31]. As already mentioned, the stress test effect in both groups in this study, should be considered adequate despite more prevalent ongoing treatment with beta-blockers in the TSC group (withheld on the day of examination) [11].

A strength of this study was the selection of both the TSC patients and the control group. The TSC patients were investigated with coronary angiography, echocardiography, chest computed tomography to exclude pulmonary embolism and CMR to exclude myocarditis. The control group was chosen from the general population often born on the same day as the TSC patients. We therefore had a good match, not just in sex, but also in age.

### Limitations

An obvious limitation of this study was the relatively small sample. Our study results did not reach statistical significance but there were numerical differences in some of the variables. With larger TSC and control groups we could have reached statistical differences.

Secondly, one of the intentions of this study was to measure the effect of mental stress on TDI, HRV and SC in TSC and to mimic the effect of an actual stressful event that triggers TSC. This poses a great challenge to the investigator, whereby there are ethical limits on the researchers and specifically the person executing the psychological stress. Given the results of the effect of the mental stress test we believe that the stress effect in this study was adequate but not enough to induce an episode of TSC.

Another limitation was the ongoing treatment with beta-blockers among many of the TSC patients. Although there was no significant difference in maximum heart rate achieved during mental stress between the groups, beta-blocker treatment may conceal a possible difference in all measurements performed in this study. This raises the question whether treatment with beta-blockers is essential in TSC patients.

## Conclusion

In this study we found no significant differences in left and right ventricular function measured with TDI or HRV between TSC patients and controls during mental stress. Moreover, there were no significant differences in SC at baseline or during mental stress. These results indicate a remarkable normalization of myocardial function over time and no remaining myocardial vulnerability to mental stress in TSC patients. However, further studies are needed for a better understanding of TDI, HRV and SC responses to mental stress in TSC patients.
